# A pilot evaluation of a computer-based psychometric test battery designed to detect impairment in patients with cirrhosis

**DOI:** 10.2147/IJGM.S140197

**Published:** 2017-09-06

**Authors:** Nicola A Cook, Jin Un Kim, Yasmin Pasha, Mary ME Crossey, Adrian J Schembri, Brian T Harel, Torben Kimhofer, Simon D Taylor-Robinson

**Affiliations:** 1 Liver Unit, Division of Digestive Health, Department of Surgery and Cancer, Imperial College London, London, UK; 2 Cogstate, Inc., New Haven, CT, USA; 3 Department of Psychology, Royal Melbourne Institute of Technology University, Melbourne, VIC, Australia; 4 Child Study Center, Yale University, New Haven, CT, USA; 5 Section of Biomolecular Medicine, Division of Computational and Systems Medicine, Department of Surgery and Cancer, Imperial College London, London, UK

**Keywords:** hepatic encephalopathy, minimal hepatic encephalopathy, cirrhosis, psychometric testing, Cogstate™, Psychometric Hepatic Encephalopathy Score

## Abstract

**Background:**

Psychometric testing is used to identify patients with cirrhosis who have developed hepatic encephalopathy (HE). Most batteries consist of a series of paper-and-pencil tests, which are cumbersome for most clinicians. A modern, easy-to-use, computer-based battery would be a helpful clinical tool, given that in its minimal form, HE has an impact on both patients’ quality of life and the ability to drive and operate machinery (with societal consequences).

**Aim:**

We compared the Cogstate™ computer battery testing with the Psychometric Hepatic Encephalopathy Score (PHES) tests, with a view to simplify the diagnosis.

**Methods:**

This was a prospective study of 27 patients with histologically proven cirrhosis. An analysis of psychometric testing was performed using accuracy of task performance and speed of completion as primary variables to create a correlation matrix. A stepwise linear regression analysis was performed with backward elimination, using analysis of variance.

**Results:**

Strong correlations were found between the international shopping list, international shopping list delayed recall of Cogstate and the PHES digit symbol test. The Shopping List Tasks were the only tasks that consistently had *P* values of <0.05 in the linear regression analysis.

**Conclusion:**

Subtests of the Cogstate battery correlated very strongly with the digit symbol component of PHES in discriminating severity of HE. These findings would indicate that components of the current PHES battery with the international shopping list tasks of Cogstate would be discriminant and have the potential to be used easily in clinical practice.

## Introduction

Hepatic encephalopathy (HE) is one of the most debilitating consequences of liver disease and is characterized as diffuse brain dysfunction caused by liver insufficiency and/or portosystemic shunting.^1^ Due to the complex pathogenesis, the neurological and/or psychiatric manifestations of the disease vary according to the extent of its severity.

The full clinical presentation manifests in patients with overt HE (OHE), which is an event that defines liver decompensation. Numerous neurological and neurocognitive domains are affected, which have protean manifestations including extrapyramidal dysfunction, asterixis, myelopathy, progressive memory impairment, disorientation for time and space, acute confusion and coma.^2^ Conversely, in minimal HE (mHE), there is none of the clinical or obvious cognitive dysfunctions of OHE and it is only detectable by psychometric testing of psychomotor speed, executive functions or neurophysical alterations.^3^ Even though mHE is blanketed by its subclinical presentation, it may have a significant impact on activities of daily living, such as impairment of the ability to drive or operate machinery safely, owing to impaired cognitive and locomotive function.^4^

Psychometric testing to identify early HE development is crucial for initiating treatment and halting disease progression to OHE, reducing the overall burden of disease to the local health care system. Furthermore, it is important to assess those with suspected mHE for the ability to drive or operate machinery safely, to minimize the personal and societal consequences of accidents.^4^

In a report by the United Kingdom Health Protection Agency, it was estimated that by 2020, there would be a significant increase in the number of people living with virally related chronic liver disease in the UK and a 10% rise in cirrhosis prevalence.^5^ With the added burden of alcohol misuse and non-alcohol-related fatty liver disease, the number of deaths from hepatic disorders is rising in the UK, compared to other parts of Europe. There is increasing pressure for quick, cost-effective ways of detecting mHE for appropriate management and treatment, thereby reducing the impact on affected individuals and their carers.

The psychometric test battery recommended by the recent European Association for the Study of the Liver/American Association for the Study of Liver Diseases guidelines is the Psychometric Hepatic Encephalopathy Score (PHES).^6^ This collection of neurocognitive tests has been designed to examine motor speed, visual perception, visual–spatial orientation, visual construction, concentration and attention and, to a lesser degree, memory.^6^ There are four different versions of each of the tests that make up the PHES to prevent patients who require repeated tests from learning and recognizing them.

### The components are

1. Number connection test A: measures concentration, mental tracking and audio visuomotor speed.
2. Number connection test B: measures concentration, mental tracking and audio visuomotor speed but with more complexity than Test A.
3. Digit symbol test (DST): measures psychomotor and visuomotor speed.
4. Line tracing test (LTT): measures visuomotor and visuospatial components for both speed and accuracy.
5. Serial dotting test (SDT): measures psychomotor speed.

The evaluation starts with a practice phase consisting of a small sample of items, so that the patient can become familiar with how each test is designed and what is required of them. The components are basic, do not require expensive equipment and can be performed in most clinical and office settings, if time allows. However, the ideal environment is a quiet room with good lighting to standardize conditions. Although feasible, completing the assessment by the bedside on a busy ward is rarely appropriate because of noise and distractions.

The PHES test has its limitations, given that it can be influenced by education, cultural background, lack of sleep, emotional upset or language difficulties.^4^ It has been adapted for use in Spain, Italy, UK and India.^7^–^10^ However, it is not used in the USA, where alternatives, such as the more-time-consuming repeatable battery for the assessment of neuropsychological status battery, which consists of 12 subtests, are used. As psychometric testing is a reimbursable cost, and to comply with the requirements of US health insurance companies, the testing is more intensive than in Europe.^11^ Although the PHES has been adopted in many centers worldwide to screen for the presence of mHE, its clinical utility has been criticized. Kircheis et al performed a placebo-controlled, clinical multicenter trial with 217 cirrhosis patients, to study the discrepancy in the expected and observed severity of HE.^12^ The study revealed that up to 50% of the patients were wrongly allocated with regard to HE severity because of the difficulty in differentiating mild OHE from mHE. Moreover, mistakes were made in analyzing and scoring the results of PHES. The contradictory results prompt recognition of other psychometric investigations of mHE.

As an alternative to paper-and-pencil–based cognitive tests, several computer-based cognitive tests have been developed, some of which have become too expensive for use in routine clinical settings. However, Cogstate™ (Cog-state Inc., New Haven, CT, USA) has developed a number of computer-based tests that are already in use for human immunodeficiency virus (HIV) patients with cognitive difficulties and have the potential to be adapted for use in mHE.^13^ The battery comprises a series of computer-based adaptations of standard neuropsychological tests that assess a range of cognitive functions, including psychomotor speed, attention, learning, and visual and verbal working memory. The battery can be customized to test the cognitive functions appropriate for certain diseases and has been validated for use in patients suffering from Alzheimer’s disease, schizophrenia, mild traumatic brain injury and HIV.^13^,^14^ The battery typically takes between 20 and 40 minutes to complete depending on the number of cognitive domains assessed and gives individuals 1) various card games on a green background, 2) maze games chasing the targets pathway around the maze and memorizing it, 3) pairing shapes and memorizing where they are with places covered on the screen by colored balls. The first and last task involve an idealized “shopping list,” which is read out to each participant at the beginning of the battery. Participants are 1) asked to recall immediately after hearing the list, and 2) then recall again at the very end of the session without hearing the list again, once they have completed the other tasks.

Normative data generated from adults from 18 to 89 years in age are available for the Cogstate tests used in this study. The data are derived from a healthy population of subjects in a series of clinical trials, research and academic studies. The baseline sessions were included in the normative database and only a single session was included for each subject. The participants were recruited from countries in North and South America, Europe, Asia and Australia.^13^

We hypothesized that a tailor-made computer battery would be more discriminant in mHE diagnosis than the standard PHES battery and that it would minimize both ceiling and floor effect of PHES, when highest and lowest scores are unable to discriminate the patient’s level of ability. Based on the hypotheses, we 1) compare individual Cogstate battery tests with PHES tests, and 2) derive a simple Cogstate test battery that can be used for detection of mHE in routine clinical practice.

## Patients and methods

The patient population consisted of 27 subjects (28 males, nine females of mean [range] age 56.9 [36–69] years) with biopsy-proven cirrhosis, attending hepatology outpatient clinics between April 2015 and April 2016 at the Imperial College Healthcare Trust, London, UK. The underlying etiology of the cirrhosis was hepatitis C infection (n=11), alcohol misuse (n=6), non-alcoholic fatty liver disease (NAFLD) (n=2), hepatitis C and NAFLD (n=1); hepatitis C and alcohol misuse (n=1), autoimmune hepatitis (n=2), hepatitis B infection (n=1), primary sclerosing cholangitis (n=1), biliary (n=1) and hemochromatosis (n=1). All subjects were able to understand and communicate in the English language. All were given a minimum of 48 h to read the patient information sheet, prior to recruitment and all gave informed, written consent, according to the guidelines adopted by the 18th World Medical Assembly in the 1964 Declaration of Helsinki on Human Rights (World Medical Assembly, 1964), and in accordance with the London – Fulham Research Ethics Committee approval (LREC reference no. 05/Q0411/71).

Exclusion criteria were recent excess alcohol consumption within the preceding 6 months, current intravenous or nasal illicit drug use, usage of psychoactive and antipsychotic drugs, known cerebrovascular disease, consumption of drinks containing caffeine 2 h prior to planned psychometric testing, or current or previous clinical evidence of OHE.

All subjects were examined clinically and had no neurological abnormalities, nor any clinical evidence of OHE. Each had blood drawn for standard liver biochemistry with Child–Pugh and United Kingdom end-stage liver disease scores being calculated to grade the functional level of cirrhosis for each patient.^15^,^16^ These were used as co-variables with PHES and Cogstate results.

All subjects underwent both the English PHES battery testing and the modified Cogstate battery tests. To avoid test bias, the order of testing was alternated: participants with an odd subject number were tested with the computer-based Cogstate battery first and the PHES battery second, and the subjects with even subject numbers were tested with the PHES battery first and the Cogstate battery second.

The PHES battery consisted of five paper-and-pencil–based tests: the number connection tests A and B (NCT-A/B), DST, SDT and LTT.^6^

The scoring of the PHES is performed by taking account of 1) the age of the subject, 2) time of test completion, and 3) accuracy of test completion with a score for healthy controls, which ranges from ≥−2 to +5. Scores of <−2 to −15 indicate varying levels of HE from mHE through to OHE.

Test results within ±1SD from the age-adjusted mean were scored with 0 points, those between −1 and −2SD were scored −1, those between −2 and −3SD beyond the mean were scored −2 points and those worse than −3SD were scored with −3 points. Results better than means + 1SD were scored +1, which allows score results to range from +6 to −18 points. The final English PHES *z* score (2 decimal points) was normalized for the UK-based population.^17^ It has taken into account ethnicity, years of education, whether subjects were educated in the UK or abroad, and weekly alcohol intake, measured in grams.

### The Cogstate battery consisted of

1. International shopping list (ISL). A list of 12 items is read out aloud to the participant three times. An ISL score (total number of correct answers across all three learning trials [ISL cor]) is derived from the number of correctly remembered items.
2. Chase test (CT). The participant has to chase the target in the grid following the exact journey the target has taken in the grid. The grid is 10×10 in size and travel up, down and side to side by one or two squares is allowed.
3. Groton Maze learning test (GMLT). The participant has to remember the 28-step pathway that is used to get from the top of the grid to the bottom. The grid is 10×10 in size. The pattern can travel up, down and side to side, but not diagonally, while travel must be only by one square at a time and participants cannot move back on the pathway. Feedback is given with visual and auditory cues to indicate whether the selected box is correct or incorrect.
4. Detection task. In this test, the playing cards all depict the same joker. The subject is asked to press the Yes key as soon as the card in the center of the screen turns face up. The software measures the speed and accuracy of each response.
5. Identification test (IDN). In this test, the playing cards are all either red or black jokers. The subject is asked whether the card displayed in the center of the screen is red. The subject responds by pressing the Yes key when the joker card is red and No when it is black. The software measures the speed and accuracy of each response.
6. One card learning task (OCL). In this test, the playing cards are identical to those found in a standard deck of 52 playing cards (without the joker cards). The subject is asked whether the card displayed in the center of the screen was seen previously in this test. The subject responds by pressing the Yes or No key. The software measures the speed and accuracy of each response.
7. One back task (ONB). In this test, the playing cards are identical to those found in a standard deck of 52 playing cards (without the joker cards). The subject is asked whether the card displayed in the center of the screen is the same as the card presented immediately previously. The subject responds by pressing the Yes or No key. Because no card has been presented yet on the first trial, a correct first response is always No. The software measures the speed and accuracy of each response.
8. Continuous paired associate learning task (CPAL). This test consists of a single amoeboid shape displayed in the center of the screen surrounded by a number of blue-filled circles. Beneath all but two of the blue spheres are amoeboid shapes, one of which matches the central display; the two remaining circles are distractors. In the exposure phase of the test all of the to-be-remembered pattern–location associations are presented on the computer screen simultaneously. After the exposure phase, a pattern in shown in the center, and the subject is required to select the peripheral location where an identical pattern is hidden beneath the blue sphere. The participant has to identify where the picture is located that matches the picture in the center target. All the pictures are covered by blue balls. Once identified, the center picture changes until each picture is paired.
9. International shopping list delayed recall (ISLR). The final task is a return to the shopping list, but this time the participant is asked to remember items on the list that were read out at the beginning of the computer battery testing. This, therefore, involves delayed recall.

The Cogstate battery was customized specifically for liver disease patients, based on those tests that were discriminant for mHE with eight of the 11 Cogstate tests used in a modified battery: 1) international shopping list test, 2) CT, 3) GMLT, 4) detection test (DET), 5) IDN, 6) OCL, 7) ONB, and 8) ISLR (Cogstate Inc.).^18^

Consequently, we selected the following variables from each Cogstate test:

CPAL: CPAL err = total number of errors
ISL: ISL cor = total number of correct answers across all three learning trials
ISLR: ISLR cor = total number of correct answers on the delayed trial
OCL: OCL acc = arcsine proportion of correct answers
ONB: ONB lmn = Log_10_ milliseconds speed of reaction for correct responses
IDN: IDN lmn = Log_10_ milliseconds speed of reaction for correct responses
DET: DET lmn = Log_10_ milliseconds speed of reaction for correct responses
CHASE: CHS mps = average moves per second over a 30-second period.

The individual patient scores for each Cogstate test were referenced to average test scores that were derived from an age-matched healthy population. This was performed automatically by the Cogstate system (Cogstate Inc.).

A Spearman’s correlation (*ρ*) analysis was performed to compare the individual subtest from the Cogstate and PHES battery. Results were visualized in a heatmap.

A final Cogstate test score (CS_total_) was then derived 1) by combining the referenced subtest scores with equal weightings, and 2) by linear modeling using a non-negative linear least-squares approach,^19^ as implemented in the R package nnls, which is available via the Comprehensive R Archive Network (www.cran.r-project.org). The optimal CS_total_ cutoff value for diagnosing mHE was then determined with the Youden’s J statistic using the dichotomous mHE classification determined by a PHES value of <−2.

## Results

A total of 27 participants completed both the PHES and the Cogstate battery tests. The median PHES score was −5 (range 0 to −13). Using a PHES total cutoff value of <−2, classified 23 patients (85%) as mHE and five patients (15%) as non-mHE.

### Equivalence (and nonequivalence) of PHES and Cogstate subtests

**Figure 1:**
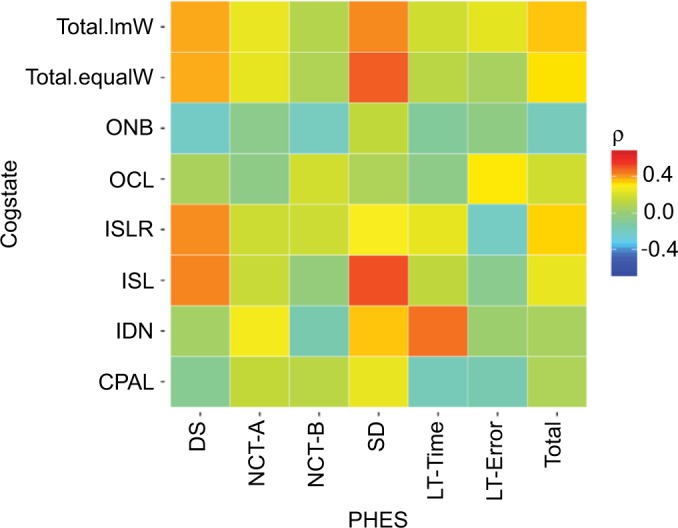
Heatmap demonstrating correlations between subtests of the PHES test (x-axis) and subtests of Cogstate battery (y-axis). **Abbreviations:** CPAL, continuous paired associated learning; DS, digital symbol task; IDN, identification test; ISL, international shopping list; ISLR, international shopping list delayed recall; LT-Error, line tracing test error; LT-Time, line tracing test time; NCT-A, number connection test A; NCT-B, number connection test B; OCL, one card learning; ONB, one back task; PHES, Psychometric Hepatic Encephalopathy Score; SD, serial dotting test; Total.lmW, Cogstate total score using non-negative linear model weighting approach; Total.equalW, Cogstate total score using equal weighting approach.

Correlation between PHES and Cogstate battery tests is visualized by the heatmap (Figure 1) and the correlation table (Table 1). The highest correlation between test scores was observed for Cogstate’s ISL and PHES’ Serial Dotting task (ρ=0.51), followed by IDN and Line Tracing Time (ρ 0.45) (Figure 1). Another high correlation magnitude was observed for both the Cogstate ISL and ISLR with PHES digital symbol task (ρ=0.42 and 0.41, respectively).

CPAL showed mainly low-magnitude correlations (maximally with PHES SDT, ρ=0.24), and similar observations were made for ONB (ρ=−0.21 with DST) and OCL (ρ=0.13 with LTT error).

Tables 2 and 3 both show a strong correlation between the ISL task from Cogstate and PHES total score. This is the only task that is present in all the independent variables in the analysis-of-covariance table, where the *P* values are <0.05.

### Cogstate battery as a tool for mHE diagnosis

**Figure 2:**
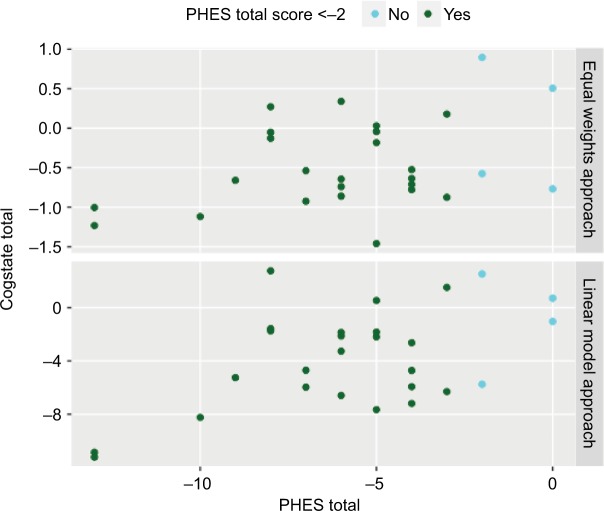
Evaluation of Cogstate battery utility in mHE diagnosis by equal weights approach (upper panel) and linear model approach (lower panel). **Note:** The PHES cutoff value for mHE was a score of <−2. **Abbreviations:** PHES, Psychometric Hepatic Encephalopathy Score; mHE, minimal hepatic encephalopathy.

For each participant, the Cogstate CPAL, IDN, ISL, ISLR, OCL and ONB scores were linearly combined to form a composite total Cogstate score. Assigning equal weightings to each subscore was not able to recover the mHE/non-mHE class membership distribution determined by a PHES total score of <−2 (Figure 2, upper panel).

However, in a non-negative linear modeling approach, individual weightings were assigned to the ONB, identification test (IDN) and the ISLR tasks (model coefficients: 1.69, 1.54 and 1.21, respectively). The CPAL task was assigned a slightly lower weighting (coefficient =0.80), and the ISL and ONB were assigned coefficients of zero, indicating that the subtests may be limited for mHE diagnosis. For participants classified as non-mHE, the final Cogstate total score ranged from 2.5 to −5.7 with a median value of −0.2. Participants classified as having mHE had on average lower Cogstate scores with a median value of −4.7, ranging from 2.7 to −11.2 (Figure 2, lower panel).

## Discussion

Our main findings indicate that certain components of the PHES battery may not be as diagnostically discriminant for mHE as had been previously reported, despite the battery being widely regarded by the International Community as the gold standard. The test battery relies on patients being able to count, follow instructions and recall the Roman alphabet. In the UK, language can be a problem if English is not a patient’s first medium, or if they are illiterate, thereby limiting its use in a multicultural setting. We controlled for this by recruiting only those patients who can maintain communication in the English language. Centers in India have addressed this issue by replacing recall of the Roman alphabet with figure connection tests as an alternative.^9^

We found that physical problems also inhibited patients from performing well in the PHES test, particularly during the line tracing task (subtest 5 of the PHES battery), where a steady hand is required as part of the test. A subject during the study with a previous arm injury subsequently found the test difficult. Similar physical limitations in patients with good cognition may lead to false-positive results.

The serial dotting (PHES subtest 4) test results of the 27 subjects varied between −2 and −3 without a range in the distribution from the total PHES score. It would seem appropriate in future to consider whether this subtest is useful in larger-scale studies with a view to developing a more streamlined four-component battery, rather than a five-component battery.

In the present study, we limited the number of people scoring the PHES test to two and both of them independently corroborated the other’s results. To ensure optimal results, we observed that the surrounding environment for cognitive testing needs careful consideration for both PHES and Cogstate, as it may have an influence on how a patient performs during the testing. The room needs to be in a quiet location, with good lighting and a comfortable temperature with mobile phones switched off.

The Cogstate battery was well received by patients: several expressed that they enjoyed the test in feedback received. Some found using the mouse to navigate around the Groton Maze and Chase the Target quite difficult because of limited experience with computers. Despite a strong correlation, the Groton Maze was removed from the test battery after the eleventh patient as it took an average of 60–90 minutes to complete per person, which is a major limitation in our aim to devise a practical battery for use in clinical scenarios. Once removed, the full modified Cogstate took an average of 20–40 minutes. CPAL err also correlated poorly, but this may be because of the level of task difficulty. Measures of psychomotor speed, CPAL err and Serial Dotting correlated poorly.

The ISL and the ISLR also had strong positive correlations with the PHES DST. These tests did not require patients to read English because the items were read to them by the person supervising the test. They had to repeat back the items they could remember. The shopping test seemed a very practical, but simple test that would be very relevant to daily activities of living, which most nursing frameworks advocate.^20^

With regard to practice effects, the PHES test may have a learning effect on repeat testing. Patients may be able to remember the components from previous testing, which could influence results.^4^,^7^–^10^ Conversely, the computer Cogstate battery was designed to be repeatable even over brief periods of retesting.

We, therefore, suggest for clinical testing that an adapted form of PHES without the Serial Dotting (four components instead of five) should be further evaluated. However, in order to adjust the PHES total score, an age-matched healthy control group would be required for validation, needing further research into this area. Modification of the current PHES battery has previously been suggested by Riggio et al, who identified that a simplified PHES without NCT-A/B was as efficient as PHES in detecting mHE and predicting subsequent occurrence of OHE.^21^

We recommend Cogstate to be used for drug studies and clinical trials, as it is a time-efficient test and the analysis is simple. The modified form is potentially suitable for clinical practice. However, the use of the ISL and the ISLR would be both easy to facilitate and be discriminatory in clinical practice, perhaps in combination with a modified four-component PHES test.

Further research should aim at increasing the sample size of subjects with an equal number of healthy controls, as well as focusing on its diagnostic efficacy within the different stages of HE. If the preliminary findings of our research are validated, then development of a “smartphone App” would be indicated, incorporating an adapted version of the ISL from the Cogstate battery and the Digit Symbol task from the PHES battery, with a simple scoring system that would be easy, quick and assessable to use in clinical practice.

## Figures and Tables

**Table 1:** Correlations between subtests of Cogstate battery and subtests of the PHES test

		Correlations				LT-Time	LT-Error	Total
		DS	NCT-A	NCT-B	SD			
CPAL	Correlation coefficient	–0.075	0.140	0.115	0.242	−0.177	−0.152	0.128
	Sig. (two-tailed)	0.711	0.487	0.566	0.224	0.377	0.448	0.525
	N	27	27	27	27	27	27	27
IDN	Correlation coefficient	0.041	0.267	−0.150	0.348	0.449*	0.020	0.099
	Sig. (two-tailed)	0.840	0.178	0.454	0.075	0.019	0.922	0.622
	N	27	27	27	27	27	27	27
ISL	Correlation coefficient	0.415*	0.156	−0.016	0.507**	0.132	−0.065	0.303
	Sig. (two-tailed)	0.031	0.437	0.938	0.007	0.512	0.749	0.124
	N	27	27	27	27	27	27	27
ISLR	Correlation coefficient	0.405*	0.171	0.169	0.288	0.237	−0.205	0.400*
	Sig. (two-tailed)	0.036	0.394	0.400	0.145	0.234	0.304	0.039
	N	27	27	27	27	27	27	27
OCL	Correlation coefficient	0.064	−0.044	0.178	0.076	−0.046	0.302	0.167
	Sig. (two-tailed)	0.753	0.826	0.373	0.708	0.818	0.126	0.405
	N	27	27	27	27	27	27	27
ONB	Correlation coefficient	−0.211	−0.058	−0.194	0.136	−0.096	−0.039	−0.157
	Sig. (two-tailed)	0.290	0.773	0.333	0.498	0.633	0.845	0.434
	N	27	27	27	27	27	27	27

**Notes:**

** Correlation is significant at the 0.01 level (two-tailed).

* Correlation is significant at the 0.05 level (two-tailed).

**Abbreviations:** CPAL, continuous paired associated learning; DS, digital symbol; IDN identification test; ISL, international shopping list; ISLR, international shopping list delayed recall; LT-Error, line tracing error; LT-Time, line tracing time; NCT-A, number connection test A; NCT-B, number connection test B; OCL, one card learning; ONB, one back task; PHES, Psychometric Hepatic Encephalopathy Score; SD, serial dotting test; Sig., significance.

**Table 2:** Model summary regression: Cogstate predicting PHES total

Model	*R*	R Square	Adjusted R square	Std. Error of the estimate	Change statistics				
					R square change	F change	*df1*	*df2*	Sig. F change
1	0.601^a^	0.361	0.170	2.815	0.361	1.886	6	20	0.133
2	0.600^b^	0.360	0.207	2.750	−0.001	0.047	1	20	0.831
3	0.597^c^	0.356	0.239	2.695	−0.004	0.125	1	21	0.727
4	0.581^d^	0.337	0.251	2.673	−0.019	0.641	1	22	0.432
5	0.563^e^	0.317	0.260	2.657	−0.020	0.702	1	23	0.411
6	0.535^f^	0.286	0.258	2.661	−0.031	1.079	1	24	0.309

**Notes:**

a Predictors: (Constant), ONB, CPAL, OCL, ISLR, ISL, IDN.

b Predictors: (Constant), ONB, OCL, ISLR, ISL, IDN.

c Predictors: (Constant), OCL, ISLR, ISL, IDN.

d Predictors: (Constant), OCL, ISL, IDN.

e Predictors: (Constant), ISL, IDN.

f Predictors: (Constant), ISL.

**Abbreviations:** CPAL continuous paired associated learning; IDN, identification test; ISL, international shopping list; ISLR, international shopping list delayed recall; OCL, one card learning; ONB, one back task; PHES, Psychometric Hepatic Encephalopathy Score; Sig. F change, significance of F change.

**Table 3:** Analysis of covariance of Cogstate (independent variable) with PHES

Model		Sum of squares	*df*	Mean square	F	Sig.
1	Regression	89.645	6	14.941	1.886	0.133^b^
	Residual	158.429	20	7.921		
	Total	248.074	26			
2	Regression	89.277	5	17.855	2.361	0.075^c^
	Residual	158.797	21	7.562		
	Total	248.074	26			
3	Regression	88.332	4	22.083	3.041	0.039^d^
	Residual	159.742	22	7.261		
	Total	248.074	26			
4	Regression	83.681	3	27.894	3.903	0.022^e^
	Residual	164.393	23	7.148		
	Total	248.074	26			
5	Regression	78.664	2	39.332	5.572	0.010^f^
	Residual	169.410	24	7.059		
	Total	248.074	26			
6	Regression	71.048	1	71.048	10.034	0.004^g^
	Residual	177.026	25	7.081		
	Total	248.074	26			

**Notes:**

b Predictors: (Constant), ONB, CPAL, OCL, ISLR, ISL, IDN.

c Predictors: (Constant), ONB, OCL, ISLR, ISL, IDN.

d Predictors: (Constant), OCL, ISLR, ISL, IDN.

e Predictors: (Constant), OCL, ISL, IDN.

f Predictors: (Constant), ISL, IDN.

g Predictors: (Constant), ISL.

**Abbreviations:** CPAL, continuous paired associated learning; IDN, identification test; ISL, international shopping list; ISLR, international shopping list delayed recall; OCL, one card learning; ONB, one back task; PHES, Psychometric Hepatic Encephalopathy Score; Sig, significance.
